# Development of the LARyNGTH score to predict central neurogenic respiratory failure in lateral medullary infarction

**DOI:** 10.3389/fneur.2026.1864132

**Published:** 2026-10-06

**Authors:** Yoon Sub Kim, So Young Park, Ji Hyun Park, Bumhee Park, Jin Soo Lee, Seong-Joon Lee

**Affiliations:** 1 Department of Neurology, Ajou University School of Medicine, Suwon, Republic of Korea; 2 Office of Biostatistics, Medical Research Collaborating Center, Ajou Research Institute for Innovative Medicine, Ajou University Medical Center, Suwon, Republic of Korea; 3 Department of Biomedical Informatics, Ajou University School of Medicine, Suwon, Republic of Korea

**Keywords:** brainstem stroke, central neurogenic respiratory failure, clinical prediction score, dysphagia screening, lateral medullary infarction, respiratory arrest

## Abstract

**Background:**

Central neurogenic respiratory failure (CNRF) is a fatal complication associated with lateral medullary infarcts (LMI). Its detection may be critical for timely intervention and to reduce the risk of mortality. This study aimed to identify the clinical predictors of CNRF in patients with LMI.

**Methods:**

This study included patients with LMI who were admitted to a university hospital between 2010 and 2022. CNRF was defined as unexpected intubations due to hypercapnia, cardiopulmonary resuscitation owing to respiratory causes, or clinical detection of CNRF. Clinical, neurological, and imaging data were also collected. Univariable and multivariable analyses were performed to identify the clinical predictors of respiratory complications.

**Results:**

Among the 214 patients, 12 (5.6%) developed CNRF. Hypertension (91.7% vs. 55.9%, *p* = 0.033) and diabetes mellitus (58.3% vs. 26.2%, *p* = 0.04) were more common in the CNRF group. Patients with CNRF exhibited higher National Institutes of Health Stroke Scale (NIHSS) scores (6.0 [4.25–7.0] vs. 2.0 [1.0–4.0], *p* < 0.001) and lower modified Gugging Swallowing Screen (GUSS) test scores (9.0 [3.5–13.75] vs. 15 [13–15], *p* = 0.014). Imaging showed a higher rate of large rostral infarcts (83.3% vs. 9.4%, *p* < 0.001) and combined extramedullary lesions (50.0% vs. 13.4%, *p* < 0.001). Multivariable analysis identified the LARyNGTH score, which included the above variables. A score >5.5 predicted CNRF with an AUROC of 0.968 (95% CI: 0.940–0.996), a sensitivity of 91.7%, a specificity of 94.6%, and an accuracy of 94.4%.

**Conclusion:**

In patients with LMI, a combined predictive model can predict CNRF and facilitate preemptive intervention.

## Introduction

Lateral medullary infarction (LMI) is a common posterior circulation syndrome caused by ischemia of the lateral medulla oblongata. Lesions on magnetic resonance imaging (MRI) can be differentiated rostro caudally as rostral, middle, and caudal, and horizontally as typical, ventral, large, lateral, and dorsal (1). Vascular pathology is most frequently seen in the vertebral artery, followed by the posterior inferior cerebellar artery. Large vessel infarction is the most common underlying cause of LMI, followed by arterial dissection and small vessel infarction (1).

Clinically, LMI presents with signs of ataxia, ipsilateral facial or contralateral body numbness, and Horner’s syndrome (2), whereas the involvement of various tracts and nuclei located in the lateral medulla causes a myriad of symptoms that can result in a wide range of neurological deficits. Failure of automatic ventilation control, also known as “Ondine’s curse,” is a rare but fatal syndrome. The incidence rate varies from 2% to 11% (1, 3–6) according to the literature.

Despite its severity, early identification of patients at risk for CNRF remains a challenge. Owing to the low event rate, clinicoradiological predictors of central respiratory failure have not been well defined. Radiologically, a study identified extensive lesions involving the rostral medulla to be associated with central respiratory failure (6). Neurologically, severe bulbar dysfunction, dysarthria, hemiparesis, and urinary retention are reported to be more frequent in patients with central respiratory failure (5, 6). Clinically, aspiration pneumonia, old age, and higher premorbid modified Rankin Scales (mRS) have been reported (5, 6). However, due to low event rates, no previous study has conducted a multivariable analysis to develop a clinical scoring system based on a combination of predictive factors that can reliably predict central respiratory failure and lead clinicians to act proactively.

Given this gap in clinical practice, our study aimed to identify predictive factors for central respiratory failure and develop a clinical scoring system to guide early intervention, using a registry database from a regional comprehensive stroke center.

## Methods

### Patient selection and classification of central respiratory failure

Patients who presented with acute ischemic stroke between January 2010 and July 2021 and were diagnosed with LMI were identified from a prospectively collected ischemic stroke registry at the university hospital (Figure 1).

**Figure 1:**
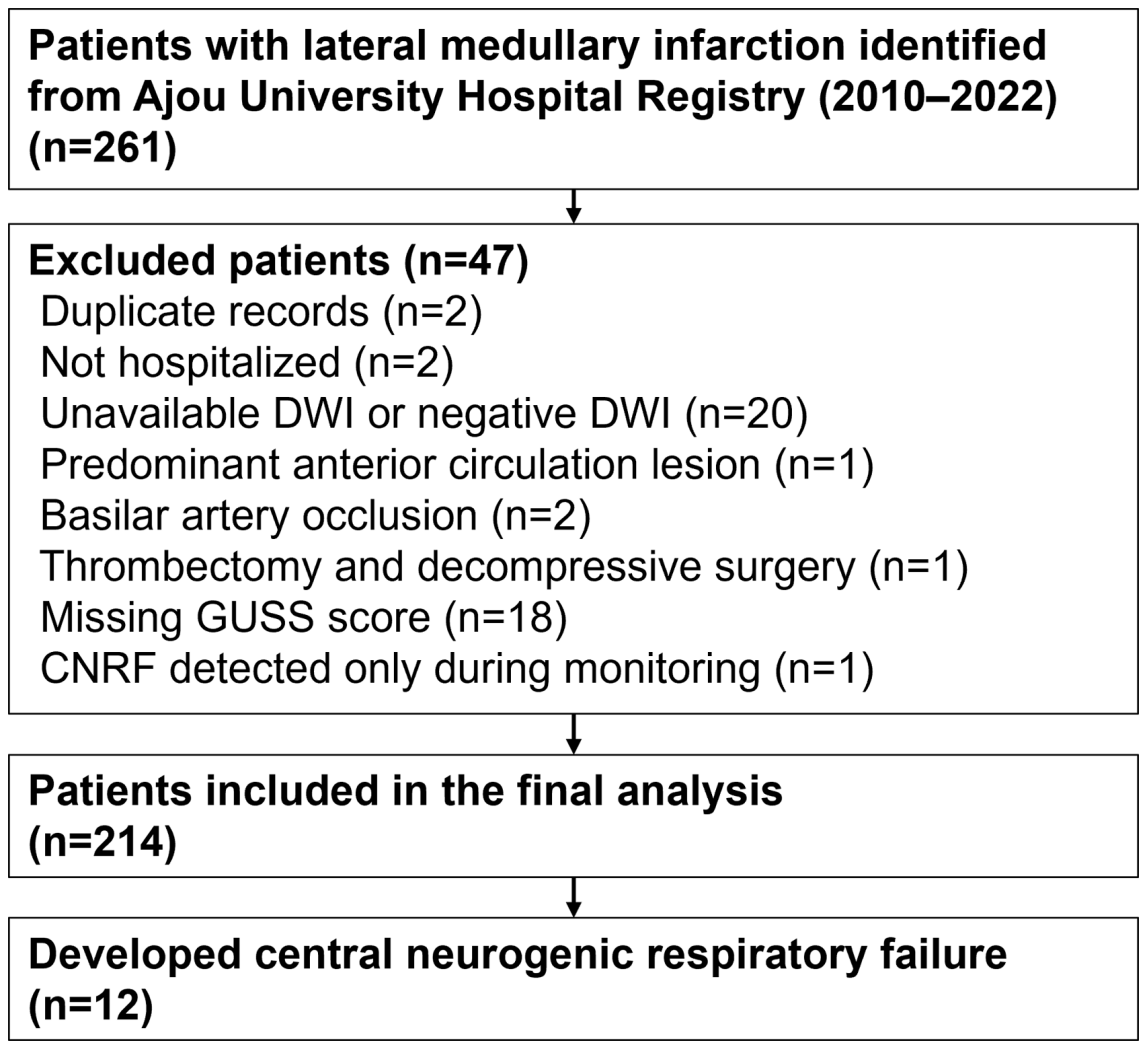
Flow diagram of patient selection. CNRF, central neurogenic respiratory failure; DWI, diffusion weighted imaging; GUSS, the Gugging Swallowing Screen test.

The exclusion criteria included the absence of diffusion restriction in the lateral medulla, predominant anterior circulation lesions, lack of major variables such as the dysphagia screening test or electrocardiography, and the presence of acute basilar artery occlusion or symptomatic hemorrhage after reperfusion treatment, as these conditions commonly result in impaired consciousness and subsequent respiratory failure, potentially through mechanisms distinct from those of CNRF associated with LMI. Patients with CNRF detection using polysomnography or intensive care unit (ICU) patient monitoring systems during admission were excluded because it is unclear whether respiratory abnormalities detected through continuous monitoring (e.g., sleep apnea) share the same pathophysiology or clinical significance as clinically overt CNRF events. Including these patients could therefore have introduced a heterogeneous population. Consequently, a small number of patients with respiratory failure were inevitably excluded from the analysis.

Cases of central respiratory failure were screened using an electronic medical record search for unexpected transfers to the critical care unit, mechanical ventilation, or use of intravenous epinephrine. Based on this search, patients with CNRF were identified by reviewing their medical records. CNRF was defined as unexpected respiratory arrest (typically due to hypercapnia) resulting in intubation and critical care management of the patient or cardiopulmonary resuscitation during the acute hospital care phase (within 2 weeks) for acute stroke. The diagnosis was established through review of clinical records by a stroke neurologist (S-JL) with 10 years of clinical experience, after exclusion of alternative causes of respiratory failure based on clinical evaluation, laboratory findings, and imaging studies. Alternative causes included aspiration pneumonia, pulmonary edema, airway obstruction (e.g., laryngospasm), sedative medications, and other identifiable cardiopulmonary or metabolic disorders.

Ethics approval was obtained from the Ajou University Hospital Institutional Review Board (AJOUIRB-DB-2025-157), and the study was performed following the ethical standards laid down in the 1964 Declaration of Helsinki and its later amendments (7). The Ajou University Hospital Institutional Review Board waived the need for obtaining patient consent.

### Clinical and radiological variables

Patient demographics, including age, sex, and vascular risk factors, were collected. Clinical severity was assessed using the serial National Institutes of Health Stroke Scale (NIHSS) scores measured upon the first evaluation by neurologists and routinely every 6 h after admission to the stroke unit by professional stroke nurses. The presence of oculomotor/oculovestibular symptoms (nystagmus or diplopia) or Horner’s syndrome (ptosis, miosis, or anhidrosis), which are generally not well captured by the NIHSS, was acquired through a review of medical records. Systolic and diastolic blood pressures measured on the day of admission combined with comorbid hypertension were included in the analysis. Dysphagia was measured upon stroke unit admission using a modified Gugging Swallowing Screen (GUSS) (8). Although the original GUSS score is composed of five points for preliminary investigation/indirect swallowing and five points each for direct swallowing of semisolids, liquids, and solid materials, we do not routinely score swallowing of solid materials and instead utilize a 15-point score. In contrast to a cutoff value of 14 points to predict aspiration risk using the full scale (9), a score of less than 10 usually indicates the need for nasogastric tube placement due to aspiration risk in the modified method. Electrocardiography (ECG) abnormalities were classified as ST or T, conduction, rhythm, or structural (S-JL) (10).

The diagnosis of LMI was established based on the complete MRI examination, including diffusion-weighted imaging (DWI) and conventional MRI sequences, together with the clinical presentation. For this study, the presence of an LMI was retrospectively confirmed on DWI by a stroke neurologist (S-JL), and lesion patterns on DWI were subsequently classified by a neurologist (YK) with 5 years of clinical experience. They were horizontally classified as typical, ventral, large, dorsal, or lateral. The lesions were classified rostro caudally as rostral, middle, or caudal (Figure 2) (1, 5). In the horizontal classification, overlapping categorization was not permitted, ensuring mutually exclusive regional assignments. In contrast, the rostro caudal classification allowed lesions to be assigned to multiple anatomical levels when appropriate, reflecting the longitudinal extension of infarcts. Using MRIcron,^1^ the lesions were carefully delineated and superimposed on a spatially unbiased atlas template of the cerebellum and brainstem to visualize the medullary infarction (11).

**Figure 2:**
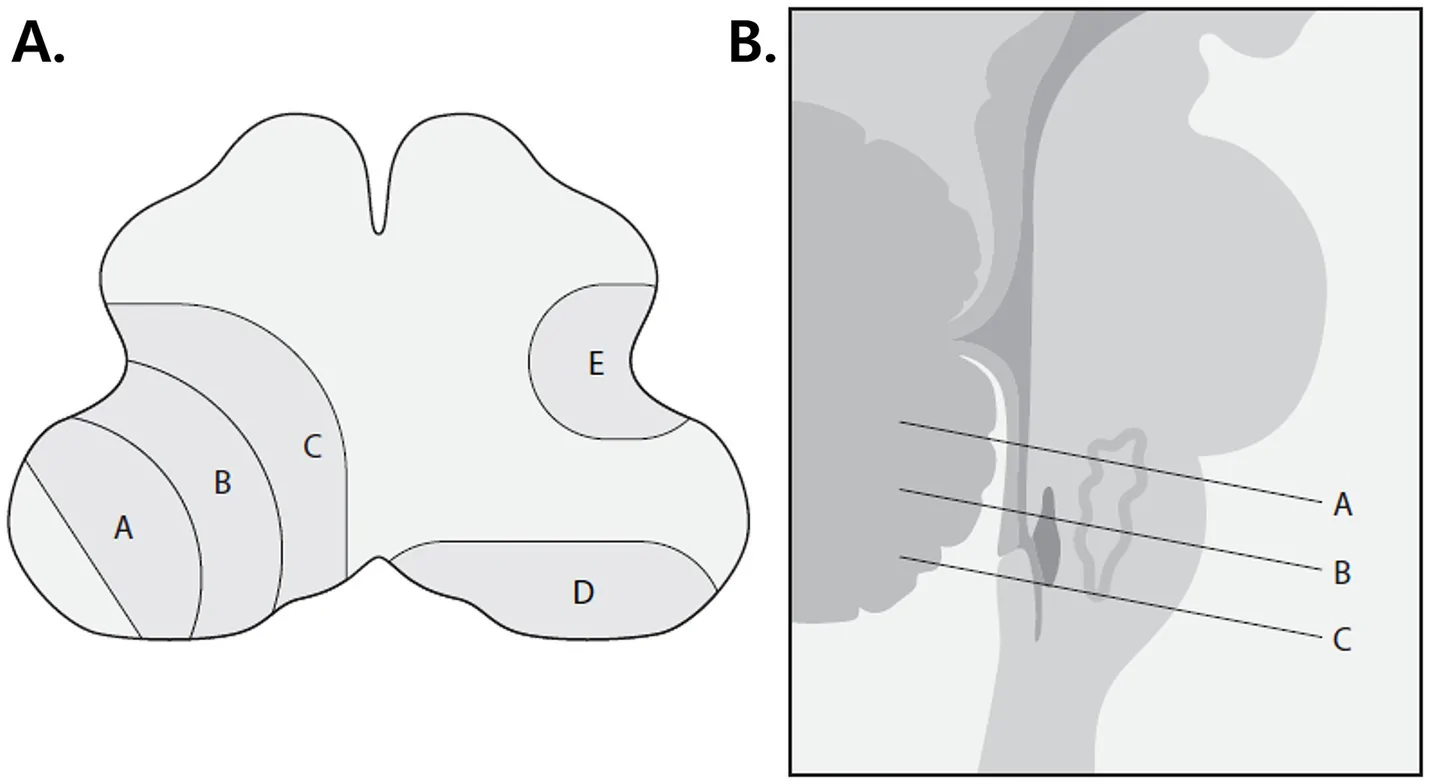
The horizontal and vertical classification of lateral medullary infarcts. **(A)** It can be horizontally classified as A + B = typical; B + C = ventral; A + B + C = large; D = dorsal, or E = lateral. **(B)** It can be also classified as A = caudal; B = middle; C = rostral (modified from Kim et al. (1)).

### Statistical analysis

Patients with LMIs were dichotomized into those with and without CNRF. Univariable analysis was performed to compare the clinical and radiological characteristics between the two groups. The identified variables were integrated into a multiple logistic regression analysis to identify the predictors of central respiratory failure and generate a clinical scoring system. For continuous variables, a cutoff point was generated by analyzing the area under the curve (AUC) of the receiver operating characteristics and sensitivity–specificity analysis. Data are presented as mean ± standard deviation, number (%), or median (interquartile range). Categorical variables were analyzed using the *χ*^2^ or Fisher’s exact test. Continuous variables were analyzed using the Student’s *t*-test. Based on multiple logistic regression analysis, a scoring system for predicting central respiratory arrest was developed. All statistical analyses were performed using R software version 4.3.3. A *p*-value less than.05 was considered statistically significant.

## Results

Among the 261 screened patients, 47 patients were excluded (duplicated data 2; not hospitalized 2; unavailable DWI or no lateral medullary infarction on DWI 20; predominant anterior circulation lesions 1; basilar artery occlusions: 2; thrombectomy and decompressive surgery 1; absent GUSS score 18; CNRF detection by monitoring 1). In a total of 214 patients with lateral medullary infarcts, CNRF occurred in 12 (5.6%) (Table 1). The CNRF group presented with a higher NIHSS score (6.0 [4.25–7.0] vs. 2.0 [1.0–4.0], *p* < 0.001) and a lower GUSS score (9.0 [3.5–13.75] vs. 15 [13–15], *p* = 0.014), suggestive of severe dysphagia. There were no differences in the onset-to-presentation days or other neurological deficits, such as oculomotor/oculovestibular signs or Horner syndrome. However, facial palsy was more frequent in the CNRF group (83.3% vs. 35.1%, *p* < 0.001). The central respiratory failure group presented more frequently with comorbid hypertension (91.7% vs. 55.9%, *p* = 0.033) and diabetes mellitus (58.3% vs. 26.2%, *p* = 0.04). There were no differences in the ECG abnormalities.

**Table 1:** Baseline clinical characteristics and imaging findings according to the occurrence of central neurogenic respiratory failure.

Variables	CNRF (+) (*N* = 12)	CNRF (−) (*N* = 202)	*p*-value
Onset to presentation, D	2.0 [1.0–3.0]	2.0 [1.0–3.0]	0.973
Initial NIHSS	6.0 [4.25–7.0]	2.0 [1.0–4.0]	<0.001
Other focal deficits			
Facial palsy	10 (83.3%)	71 (35.1%)	<0.001
Oculomotor/oculovestibular	8 (66.7%)	89 (44.1%)	0.126
Horner syndrome	5 (41.7%)	79 (39.1%)	0.860
GUSS score	9.0 [3.5–13.75]	15.0 [13.0–15.0]	0.014
Age	66 ± 14	57 ± 15	0.051
Sex, male	10 (83.3%)	148 (73.3%)	0.736
HTN	11 (91.7%)	113 (55.9%)	0.033
DM	7 (58.3%)	53 (26.2%)	0.04
Dyslipidemia	5 (41.7%)	55 (27.2%)	0.279
ECG abnormality			
Any	4 (33.3%)	75 (37.1%)	0.791
ST&T abnormality	2 (16.7%)	44 (21.8%)	0.675
Conduction abnormality	2 (16.7%)	40 (19.8%)	0.790
Rhythm abnormality	2 (16.7%)	10 (5.0%)	0.087
Structural abnormality	1 (8.3%)	30 (14.9%)	0.533
DWI lesion			
Horizontal classification			
Dorsal	0 (0.0%)	6 (3.0%)	0.545
Large	10 (83.3%)	57 (28.2%)	<0.001
Lateral	0 (0.0%)	40 (19.8%)	0.087
Typical	1 (8.3%)	48 (23.8%)	0.217
Ventral	1 (8.3%)	34 (16.8%)	0.439
Rostro-caudal classification			
Caudal	0 (0.0%)	55 (27.2%)	0.036
Middle	4 (33.3%)	82 (40.6%)	
Rostral	12 (100.0%)	73 (36.1%)	<0.001
Large rostral infarcts	10 (83.3%)	19 (9.4%)	<0.001
Combined extramedullary lesions	6 (50.0%)	27 (13.4%)	<0.001
Right sided	4 (33.3%)	97 (47.8%)	0.330
VA steno-occlusive lesion	9 (75.0%)	130 (64.0%)	0.440

CNRF, central neurogenic respiratory failure; NIHSS, National Institutes of Health Stroke Scale; GUSS, the Gugging Swallowing Screen test; HTN, hypertension; DM, diabetes mellitus; ECG, electrocardiography; VA, vertebral artery.

In the analysis of DWI lesions for horizontal classification, there was a significantly higher rate of large lesions in the central respiratory failure group (83.3% vs. 28.2%, *p* < 0.001). According to the rostro-caudal classification, rostral lesions were more frequently observed in the central respiratory failure group (100.0% vs. 36.1%; *p* < 0.001). A large rostral infarct pattern was evaluated separately, with a higher ratio in the central respiratory failure group (83.3% vs. 9.4%, *p* < 0.001). The presence of combined extramedullary lesions was also higher in the respiratory failure group (50.0% vs. 13.4%, *p* < 0.001). There were no significant differences between the laterality of the medullary lesion (right vs. left) and steno-occlusive vertebral artery lesions. Figure 3 shows the superimposed lesion plots of the 12 patients with CNRF, showing the predominant involvement of large rostral lesions (for convenience, and the insignificance of lesion laterality, all lesions are depicted in the left hemisphere).

**Figure 3:**
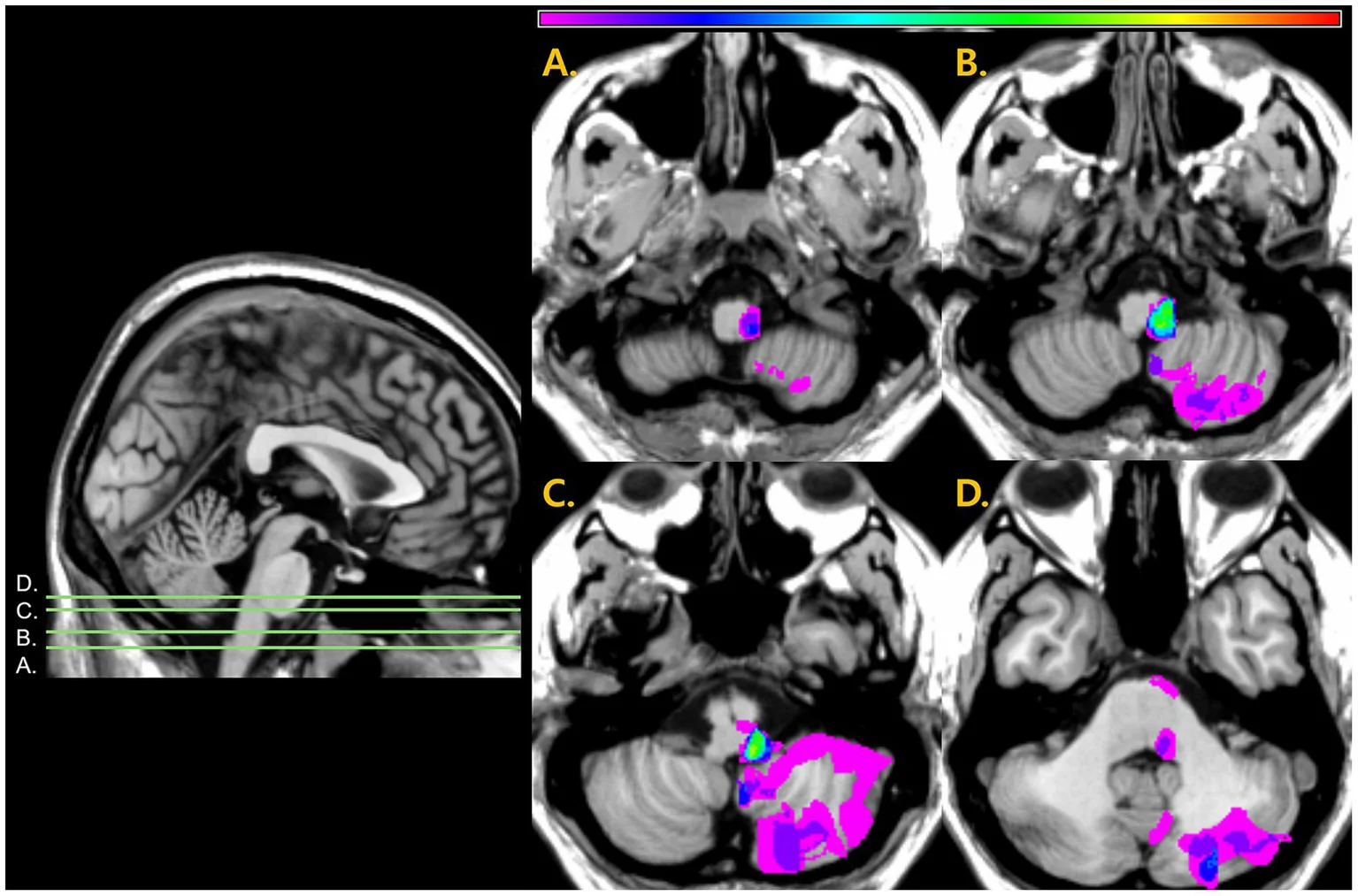
Diffusion lesion heatmaps of central neurogenic respiratory failure. **(A)** Caudal medulla (*Z* = 14); **(B)** Middle medulla (*Z* = 19); **(C)** Rostral medulla (*Z* = 26); **(D)** Caudal pons.

Cutoff values were generated for significant continuous variables, including the initial NIHSS and GUSS scores. Owing to the small number of outcome events, conventional logistic regression resulted in unstable estimates. Therefore, we applied Firth’s penalized likelihood method to reduce the small sample bias and obtain more stable estimates. When significant variables identified through univariable analyses were included, large rostral lesions (ß: 2.95, OR: 19.13, 95% CI [4.50–118.38]), NIHSS>4 (ß: 2.14, OR: 8.48, 95% CI [1.76–59.75]), GUSS<10 (ß: 1.97, OR: 7.15, 95% CI [1.53–45.00]), HTN (ß: 2.35, OR: 10.50, 95% CI [1.58–150.30]) were significantly predictive of CNRF (Table 2) while the variables age, facial palsy, comorbid diabetes mellitus, and infarction at other locations were not significant in the multivariable model. Based on this model, the LARyNGTH score (ranging from 0 to 9) was developed using Firth logistic regression estimates, assigning points for each risk factor as follows: large rostral lesions, 3 points; NIHSS score greater than 4, 2 points; GUSS score < 10, 2 points; and hypertension, 2 points. Using this score, an AUROC curve was generated with an AUC of 0.968 (95% CI, 0.940–0.996; *p* < 0.001). The optimal cutoff value generated using the Youden index was 5.5, indicating that the presence of 3 out of the 4 risk factors was highly predictive of CNRF, with a sensitivity of 91.67% (95% CI:61.52–99.79), specificity of 94.55% (95% CI: 90.47–97.25), positive predictive value of 50.00% (95% CI: 28.22–71.78), negative predictive value of 99.48% (95% CI: 97.13–99.99), and an accuracy of 94.39% (95% CI: 90.41–97.07) (Figure 4).

**Table 2:** Multivariable analysis of clinical and imaging variables associated with central respiratory failure.

Variable	Event occurrence no. of occurrence/total no. (%)	*β*	OR (95% CI)	*p*-value
Large rostral				
No	2/185 (1.1)	Ref.	Ref.	
Yes	10/29 (34.5)	2.95	19.13 (4.50, 118.38)	<0.001
NIHSS				
Normal or mild (0–4)	3/171 (1.8)	Ref.	Ref.	
Moderate or worse (≥5)	9/43 (20.9)	2.14	8.48 (1.76, 59.75)	0.007
GUSS				
Mild or moderate (≥10)	5/174 (2.9)	Ref.	Ref.	
Severe (0–9)	7/40 (17.5)	1.97	7.15 (1.53, 45.00)	0.012
HTN				
No	1/90 (1.1)	Ref.	Ref.	
Yes	11/124 (8.9)	2.35	10.50 (1.58, 150.30)	0.012

Firth’s penalized likelihood method was used. The initial model included large rostral lesions, NIHSS score, GUSS score, hypertension, age, facial palsy, diabetes mellitus, and infarction at other locations.

NIHSS, National Institutes of Health Stroke Scale; GUSS, the Gugging Swallowing Screen test; HTN, hypertension.

**Figure 4:**
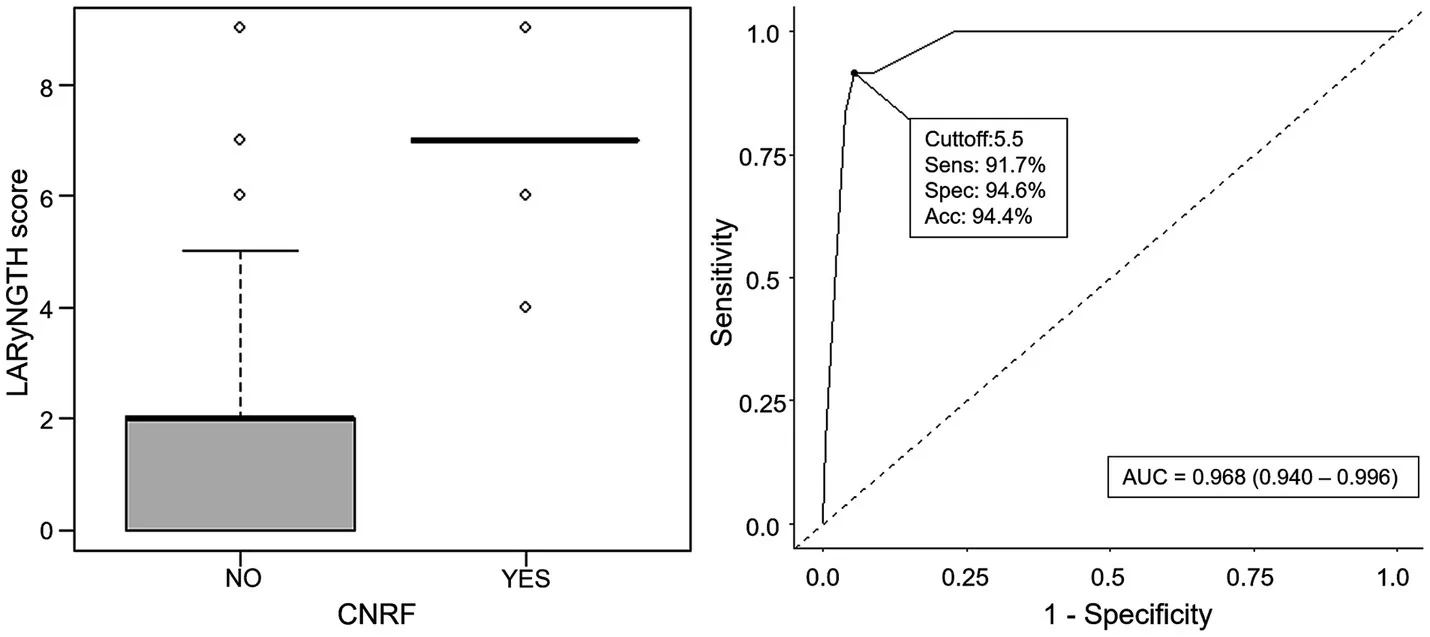
Area under the receiver operating characteristics curve (AUROC) for the LARyNGTH score and its predictive ability for central neurogenic respiratory failure. CNRF, central neurogenic respiratory failure; AUC, Area under the curve.

## Discussion

The results of the current study showed that CNRF, a devastating consequence of lateral medullary infarction, can be effectively predicted using the novel LARyNGTH scoring system. The score is a combination of DWI lesion anatomical distribution, neurological severity stratified by the NIHSS, dysphagia severity stratified by the GUSS score, and comorbid hypertension, all of which can be routinely obtained during the hospitalization of patients with ischemic stroke.

Previous reports have attempted to predict respiratory failure and investigated factors similar to those in our study. In 102 Japanese patients with LMI, fatal respiratory failure was associated with higher premorbid mRS scores, pulmonary disease, severe dysphagia, dysarthria, ipsilateral hemiparesis, and urinary retention. There were no significant differences in infarct locations (5). In a cohort of 71 Slovenian patients, respiratory failure was associated with extensive lesions involving the open medulla, progression of bulbar symptoms, aspiration pneumonia, and older age (6). A more recent study performed in China involving 435 patients with medullary infarct identified specific lesion patterns, such as large and typical infarctions in LMI patients, or long lesions, particularly those with bilateral infarctions, as V-shape or heart-shape on MRI in MMI patients as predictive of respiratory failure (12). However, these studies failed to identify the independent predictive ability of variables in multivariable analysis. Compared to these studies, the results of the current study identified key predictive factors of CNRF and presented a novel scoring system with an excellent performance.

Normal respiratory rhythm is believed to be generated by two oscillators: the parafacial respiratory group and the pre-Bötzinger complex (13). These anatomic structures are located more rostral than the ventral respiratory group or the high cervical spinal cord respiratory group. Furthermore, dysphagia, which is thought to be associated with involvement of the nucleus ambiguus, which is also located in the rostral medulla, points towards a more rostral location associated with CNRF (14). In terms of horizontal lesion distribution, large lesions involving the rostral medulla involve both the dorsal and ventral sides of the lateral medulla. Unlike the cardiac system, the respiratory rhythm is not believed to be produced by a homogeneous population of pacemaker cells. In contrast, it is believed to be generated by the core rhythm-generating neuronal circuitry composed of the pre-Bötzinger complex, Bötzinger complex, and pattern-generating neuronal elements of the ventral and dorsal respiratory groups (15). In this regard, a larger lesion involving multiple respiratory centers is more likely to be predictive of CNRF. Evidence suggests that chronic activation of peripheral chemoreceptors contributes to the development of hypertension (16). The association between hypertension and CNRF may suggest this mechanism. However, the exact underlying mechanism remains to be elucidated.

The current study did not find extramedullary lesions to be predictive of CNRF in the multivariable analysis. This may be because patients with basilar artery occlusions or large infarcts causing herniation were excluded from the current study. This supports the association of respiratory complex compromise as the cause of most CNRF encountered in LMI. Furthermore, contrary to our initial expectations, ECG abnormalities were not associated with CNRF. This also emphasizes that respiratory failure in these patients indicates a central mechanism rather than an association with conventional cardiopulmonary risk factors.

The current study opens the possibility of early preemptive intervention for patients with CNRF, as nearly half of patients with CNRF face grave outcomes with high mortality (12), and a significant number of patients are discharged with tracheostomy (6). Although respiratory failure usually tends to occur during the acute phase of admission, it may sometimes occur in the chronic phase (17). Furthermore, we cannot be sure about the duration or recovery rate of the CNRF, as it may persist in some patients beyond the acute phase, necessitating continued ventilatory support (18). Therefore, the LARyNGTH score may provide a framework for prospective patient identification and clinical trials to improve patient outcomes in this grave population. Possible interventions include end-tidal carbon dioxide monitoring, noninvasive ventilation (18), or diaphragmatic pacing (19).

Our study has several limitations. First, although CNRF is an uncommon complication of LMI, the number of patients with CNRF were small, which may have limited the statistical power to detect associations and reduce the generalizability of our findings. Second, because only patients with complete data for all major variables were included, some patients with CNRF may have been excluded, especially if respiratory arrest occurred very early, such as before the presentation to the hospital or emergency department and prior to dysphagia screening. Third, although lesion mapping was performed using specialized tools, the horizontal and vertical classifications used to generate the predictive score were visually graded, leaving the possibility of rater-dependent bias. Finally, although the current study generated the LARyNGTH score with excellent predictive ability for CNRF, its function needs to be confirmed in a validation dataset or prospective database. Therefore, these limitations call for the confirmation of these findings in a large multicenter clinical cohort and warrant future clinical trials.

In conclusion, we developed the LARyNGTH score, which appears to have excellent predictive ability for detecting CNRF in patients with LMI. CNRF seems to be most strongly associated with large rostral medullary brain lesions involving two respiratory oscillators: the parafacial respiratory group and the preBötzinger complex, combined with neurological severity using the NIHSS, dysphagia scores, and comorbid risk factor. This score can guide clinicians in the preemptive management of this potentially serious complication.

## Data Availability

Data supporting the findings of this study are available from the corresponding author upon reasonable request.

## Footnotes

1
www.mccauslandcenter.sc.edu/mricro/mricron
